# Diagnostic Performance of Sex-Specific Modified Metabolite Patterns in Urine for Screening of Prediabetes

**DOI:** 10.3389/fendo.2022.935016

**Published:** 2022-07-14

**Authors:** Zaifang Li, Yanhui Zhang, Miriam Hoene, Louise Fritsche, Sijia Zheng, Andreas Birkenfeld, Andreas Fritsche, Andreas Peter, Xinyu Liu, Xinjie Zhao, Lina Zhou, Ping Luo, Cora Weigert, Xiaohui Lin, Guowang Xu, Rainer Lehmann

**Affiliations:** ^1^ CAS Key Laboratory of Separation Science for Analytical Chemistry, Dalian Institute of Chemical Physics, Chinese Academy of Sciences, Dalian, China; ^2^ University of Chinese Academy of Sciences, Beijing, China; ^3^ Liaoning Province Key Laboratory of Metabolomics, Dalian Institute of Chemical Physics, Chinese Academy of Sciences, Dalian, China; ^4^ School of Computer Science & Technology, Dalian University of Technology, Dalian, China; ^5^ Department for Diagnostic Laboratory Medicine, Institute for Clinical Chemistry and Pathobiochemistry, University Hospital Tübingen, Tübingen, Germany; ^6^ Institute for Diabetes Research and Metabolic Diseases (IDM) of the Helmholtz Zentrum München at the University of Tuebingen, Tuebingen, Germany; ^7^ German Center for Diabetes Research (DZD), Tübingen, Germany; ^8^ Internal Medicine 4, University Hospital Tuebingen, Tuebingen, Germany

**Keywords:** prediabetes, urine, sex-specific biomarkers, modified metabolite patterns, metabolomics, screening

## Abstract

**Aims/Hypothesis:**

Large-scale prediabetes screening is still a challenge since fasting blood glucose and HbA_1c_ as the long-standing, recommended analytes have only moderate diagnostic sensitivity, and the practicability of the oral glucose tolerance test for population-based strategies is limited. To tackle this issue and to identify reliable diagnostic patterns, we developed an innovative metabolomics-based strategy deviating from common concepts by employing urine instead of blood samples, searching for sex-specific biomarkers, and focusing on modified metabolites.

**Methods:**

Non-targeted, modification group-assisted metabolomics by liquid chromatography–mass spectrometry (LC-MS) was applied to second morning urine samples of 340 individuals from a prediabetes cohort. Normal (*n* = 208) and impaired glucose-tolerant (IGT; *n* = 132) individuals, matched for age and BMI, were randomly divided in discovery and validation cohorts. ReliefF, a feature selection algorithm, was used to extract sex-specific diagnostic patterns of modified metabolites for the detection of IGT. The diagnostic performance was compared with conventional screening parameters fasting plasma glucose (FPG), HbA_1c_, and fasting insulin.

**Results:**

Female- and male-specific diagnostic patterns were identified in urine. Only three biomarkers were identical in both. The patterns showed better AUC and diagnostic sensitivity for prediabetes screening of IGT than FPG, HbA_1c_, insulin, or a combination of FPG and HbA_1c_. The AUC of the male-specific pattern in the validation cohort was 0.889 with a diagnostic sensitivity of 92.6% and increased to an AUC of 0.977 in combination with HbA_1c_. In comparison, the AUCs of FPG, HbA_1c_, and insulin alone reached 0.573, 0.668, and 0.571, respectively. Validation of the diagnostic pattern of female subjects showed an AUC of 0.722, which still exceeded the AUCs of FPG, HbA_1c_, and insulin (0.595, 0.604, and 0.634, respectively). Modified metabolites in the urinary patterns include advanced glycation end products (pentosidine-glucuronide and glutamyl-lysine-sulfate) and microbiota-associated compounds (indoxyl sulfate and dihydroxyphenyl-gamma-valerolactone-glucuronide).

**Conclusions/Interpretation:**

Our results demonstrate that the sex-specific search for diagnostic metabolite biomarkers can be superior to common metabolomics strategies. The diagnostic performance for IGT detection was significantly better than routinely applied blood parameters. Together with recently developed fully automatic LC-MS systems, this opens up future perspectives for the application of sex-specific diagnostic patterns for prediabetes screening in urine.

## Introduction

In the 21st century, prediabetes reached a pandemic scale with an estimated prevalence of >860 million people worldwide in 2021 (1). This metabolic state lasts for many years before the manifestation of type 2 diabetes (T2D). This state is not only pathologic, but also particularly amenable for treatment. However, subjects with prediabetes are often not aware of the disease (2) because the only reliable diagnostic option at this stage is the oral glucose tolerance test (OGTT), which is laborious and therefore impracticable for population-based screening approaches. On the other hand, early detection of individuals in the prediabetic phase can open promising perspectives to prevent, or at least greatly delay the onset of T2D by appropriate interventions (3).

The recommended and frequently applied screening parameters for the diagnosis of T2D, i.e., fasting plasma glucose (FPG) and hemoglobin A1c (HbA_1c_), show only a low diagnostic sensitivity in the prediabetic state in comparison to the OGTT. Data from screening studies in Europe, Asia, and America reported that approximately 50% of people in the prediabetic state will miss FPG or HbA_1c_ application (4–9). Barry and colleagues concluded from their meta-analysis that HbA_1c_ is neither sensitive nor specific for detecting prediabetes, and that a combination with FPG does not considerably improve the diagnostic performance (5). Consequently, up to now, no practicable and reliable diagnostic tool is available, other than the OGTT.

Numerous analytical approaches have been employed to detect novel biomarkers for the diagnosis of prediabetes, including more than 60 metabolomics-driven investigations recently summarized in two reviews (10, 11). Most of these metabolomics-based studies used blood samples, and the results were dominated by a few metabolite classes, namely, branched-chain and other amino acids, acylcarnitines, lysophospholipids, phosphatidylcholines, free fatty acids, and triglycerides (10, 11). Although the results showcased altered metabolic pathways in the prediabetic state and have been the basis for novel hypotheses on pathomechanisms or therapeutic approaches, no biomarker profile has made it into routine disease screening up to now.

In the present study, we took a diagnostic biomarker discovery approach that differed in four essential aspects from common metabolomics biomarker discovery studies in the diabetes field. (A) Urine was used as sample material (in view of easy, non-invasive sample collection). Up to the present, urine was very seldom used as sample material in mass spectrometric metabolomics approaches for the discovery of biomarkers in metabolic diseases (12–14). (B) Considering the individual diversity of this polygenetic disease, we aimed to discover a pattern of several metabolites instead of the usual one or few metabolite biomarkers. (C) We focused on sex-specific diagnostic patterns. (D) Only metabolites containing modifying groups were considered. The rationale to apply our novel metabolomics strategy for the untargeted profiling of modified metabolites (15) was that modified metabolites are not only less covered by common metabolomics approaches but also less represented in common metabolite databases (15, 16), which suggests that this group of metabolites could have an added value for diagnostic biomarker patterns.

## Materials and Methods

### Study Design and Study Samples

**Figure 1:**
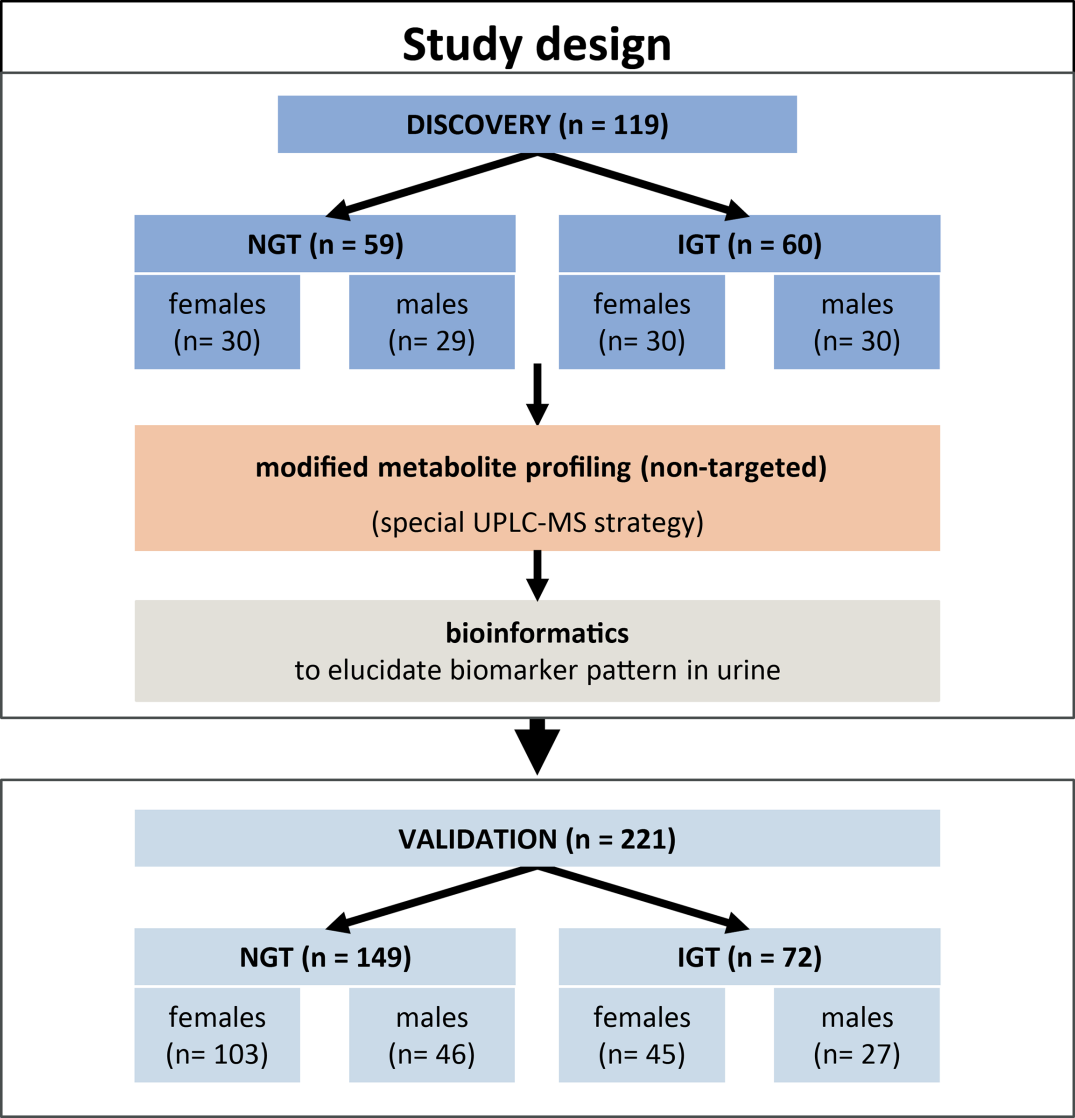
Overview of the study design and cohorts for the discovery and validation of a diagnostic pattern in second morning urine for prediabetes screening.

An overview of the general study design is given in **Figure 1**. Samples collected at baseline from a total of 340 study participants of the TULIP prediabetes cohort, recruited and studied at the University Hospital in Tuebingen, Germany, were investigated in this study. Inclusion criteria for the TULIP cohort were one or more of the following: a family history of T2D, BMI > 27 kg/m^2^, impaired glucose tolerance, and previous diagnosis of gestational diabetes. Individuals with manifest diabetes (blood glucose at 120 min >11.0 mmol/L), kidney dysfunction (GFR < 50 ml/min), liver disease, systemic infection, or critical mental illness were excluded. Informed written consent was obtained from all participants, and the Ethics Committee of the University of Tuebingen approved the protocol (ref. 422/2002) according to the Declaration of Helsinki of 1964 and its later amendments. The cohort for biomarker discovery comprised 119 individuals [59 normal glucose tolerant (NGT) and 60 impaired glucose tolerant (IGT)] selected at random. For validation of the discovered biomarker pattern, samples of 221 individuals (149 NGT and 72 IGT) were analyzed (**Table 1**). Second morning urines and blood samples were collected after an overnight fast. Thereafter, each individual performed an OGTT. Diagnostic criteria for IGT was blood glucose >7.78 mM at 120 min OGTT.

**Table 1:** Anthropometric and clinical characteristics of normal glucose-tolerant (NGT) and impaired glucose-tolerant (IGT) individuals of the cohorts of the study.

Characteristics	Discovery cohort (*n* = 119)						Validation cohort (*n* = 221)					
	All		Male		Female		All		Male		Female	
	NGT	IGT	NGT	IGT	NGT	IGT	NGT	IGT	NGT	IGT	NGT	IGT
***n***	59	60	29	30	30	30	149	72	46	27	103	45
**Age (years)**	55.7 ± 9.7	56.1 ± 9.5	56.2 ± 10.8	55.9 ± 7.5	55.2 ± 8.7	56.4 ± 11.3	55.2 ± 11.5	56.2 ± 10.9	56.1 ± 12.7	59.9 ± 9.7	54.7 ± 10.9	54 ± 11.1
**BMI (kg/m^2^)**	31.8 ± 5.2	32.5 ± 6.9	32.0 ± 4.8	33.0 ± 6.0	31.5 ± 5.6	32.0 ± 7.7	30.9 ± 5.6	31.5 ± 5.6	30.3 ± 4.2	31.7 ± 4.6	31.2 ± 6.1	31.3 ± 6.1
**Fasting glucose (mM)**	5.58 ± 0.54	6.04 ± 0.61*	5.56 ± 0.43	6.05 ± 0.60*	5.61 ± 0.63	6.04 ± 0.63*	5.62 ± 0.56	5.85 ± 0.69*	5.65 ± 0.52	5.83 ± 0.69	5.61 ± 0.57	5.86 ± 0.70*
**120 min glucose (mM)**	6.26 ± 0.91	9.29 ± 0.83*	6.29 ± 0.93	9.27 ± 0.86*	6.23 ± 0.90	9.31 ± 0.81*	6.00 ± 1.09	9.01 ± 1.17*	6.04 ± 1.22	8.99 ± 1.20*	5.97 ± 1.04	9.02 ± 1.17*
**Fasting insulin (pmol/L)**	75 ± 32	101 ± 58*	79 ± 28	95 ± 52	72 ± 36	106 ± 65*	78 ± 45	103 ± 65*	81 ± 43	101 ± 71	77 ± 47	104 ± 62*
**HbA_1c_ (mmol/mol)**	39 ± 4	40 ± 4*	38 ± 4	40 ± 4	40 ± 3	41 ± 5	39 ± 4	41 ± 4*	38 ± 4	40 ± 5*	39 ± 4	41 ± 4*
**Urine creatinine (mg/dl)**	128 ± 63	154 ± 83	143 ± 53	180 ± 92	114 ± 69	129 ± 65	131 ± 71	117 ± 53	158 ± 84	127 ± 56	118 ± 61	110 ± 50
**Urine protein (g/L)**	0.09 ± 0.05	0.12 ± 0.11	0.08 ± 0.03	0.13 ± 0.11	0.09 ± 0.07	0.11 ± 0.11	0.10 ± 0.14	0.08 ± 0.04	0.13 ± 0.23	0.08 ± 0.04	0.09 ± 0.07	0.08 ± 0.05

All, female and male subjects in one cohort; NGT, normal glucose tolerant; IGT, impaired glucose tolerant; *p < 0.05 (IGT vs. NGT; Mann–Whitney U test).

Urine samples for metabolomics analysis were stored at once at −80°C. Glucose, insulin, urinary total protein, and creatinine were analyzed immediately on ADVIA clinical chemistry and Centaur immunoanalyzer systems (Siemens Healthineers, Germany). HbA_1c_ was quantified after blood drawing by HPLC (Tosoh biosciences, Japan).

### Sample Preparation

Urine was thawed on ice and vortexed, then internal standard mix (carnitine C2:0-d_3_, carnitine C6:0-d_3_, carnitine C10:0-d_3_, leucine-d_3_, phenylalanine-d_5_, tryptophan-d_5_, cholic acid-2,2,4,4-d_4_, chenodeoxycholic acid-2,2,4,4-d_4_, leucine enkephalin, indoxyl sulfate-[^13^C_6_], L-valine-d_8_, sodium-2-hydroxybutyrate-2,3,3-d_3_, and L-4-hydroxyphenyl-d_4_-alanine) was added (v/v, 1:10), samples were vortexed again and centrifuged at 18,000 × *g* (4 °C for 10 min), and 100 μL of the supernatant was subsequently evaporated and stored at −80 °C. Before mass spectrometric analysis, samples were dissolved in 300 µL of H_2_O:methanol (v/v, 3:1).

### Non-Targeted, Modifying Group-Assisted Metabolomics

The coverage of common metabolomics approaches was broadened applying our recently published analytical strategy that focuses on modified metabolites (15). The published approach was applied with one relevant variation. To improve the coverage in urine, a UHPLC-column with a pentafluorophenylpropyl (PFPP) stationary phase having multiple selectivity mechanisms including that for polar substances was used instead of an octyldecyl-silane stationary phase. A total of 15 different types of modifications were profiled in this study (**Supplementary Table 1**). In brief, an ACQUITY Ultra Performance LC system (UPLC, Waters, Milford, U.S.A.) equipped with a Discovery HS F5-3 column (2.1 mm × 150 mm, 3 μm, Sigma Aldrich, St. Louis, USA) hyphenated to a Triple TOF 5600+ (AB SCIEX, Framingham, USA) was used for the non-targeted profiling of modified metabolites. Mobile phase A was water containing 0.1% formic acid, and mobile phase B contained acetonitrile with 0.1% formic acid. The flow rate was set as 0.35 mL/min and column temperature was set to 40 °C. Gradient elution was started with 2% B, increased to 10% B at 5 min, 40% B at 15 min, and 98% B at 20 min, then switched back to 2% B within 0.5 min and kept for 4.5 min. The injection volume was 10 µL. Data acquisition was performed in full MS-IDA scan mode with a mass range setting of *m/z* 50–1000 Da and 30–1000 Da, respectively. Accumulation time for the full scan and for the MS/MS acquisition were 0.25 s and 0.03 s, respectively. Cycle time was 0.75 s. Declustering potential was set at 90. The temperature of the electrospray ion source was set at 500 °C and 450 °C for positive and negative ion mode, respectively, and a spray voltage at 5,500 V for positive and 4,500 V for negative ion mode. Every tenth sample was followed by a quality control (QC) analysis of pooled urine.

For modified metabolite profiling, collision energies of 15 V, 30 V, and 45 V were applied as described in detail in (15), and MS/MS fragmentation patterns of the 15 most intense ions in full scan were acquired. Curtain gas, gas 1, and gas 2 were 35, 50, and 50 psi, respectively.

### Annotation of Biomarkers

Annotation of the modified metabolite features was performed according to our recently published strategy (15) with slight modifications of using five instead of three databases (OSI-SMMS, HMDB, Metlin, MyCompoundID, and Compound Discovery 3.0). Since Compound Discovery 3.0 database searches work only with high-resolution MS/MS spectra data from Thermo Fisher Scientific instruments for this purpose, MS/MS spectra from a QC sample were acquired on a QE-HF mass spectrometer (ESM Method). Furthermore, enzymatic cleavage experiments (16, 17) were applied to confirm two major types of modifications (glucuronidation and sulfation). In brief, aliquots of pooled urines were treated with either sulfatase or β-glucuronidase from Type H-2 Helix pomatia (Sigma-Aldrich; St. Louis, U.S.A.) and subsequently analyzed by LC-MS and compared with data from untreated samples. Enzymolysis buffer containing 500 μL of 0.15 M sodium acetate buffer (pH 4.3), 2.0 mg of L-ascorbic acid, and 5 μL of β-glucuronidase (≥85,000 U/ml) or sulfatase solution (≥2,000 U/ml) was mixed with the same volume of urine and incubated at 37 °C for 24 h. Control samples were processed in the same way but without the addition of β-glucuronidase or sulfatase.

### Data Analysis

**Figure 2:**
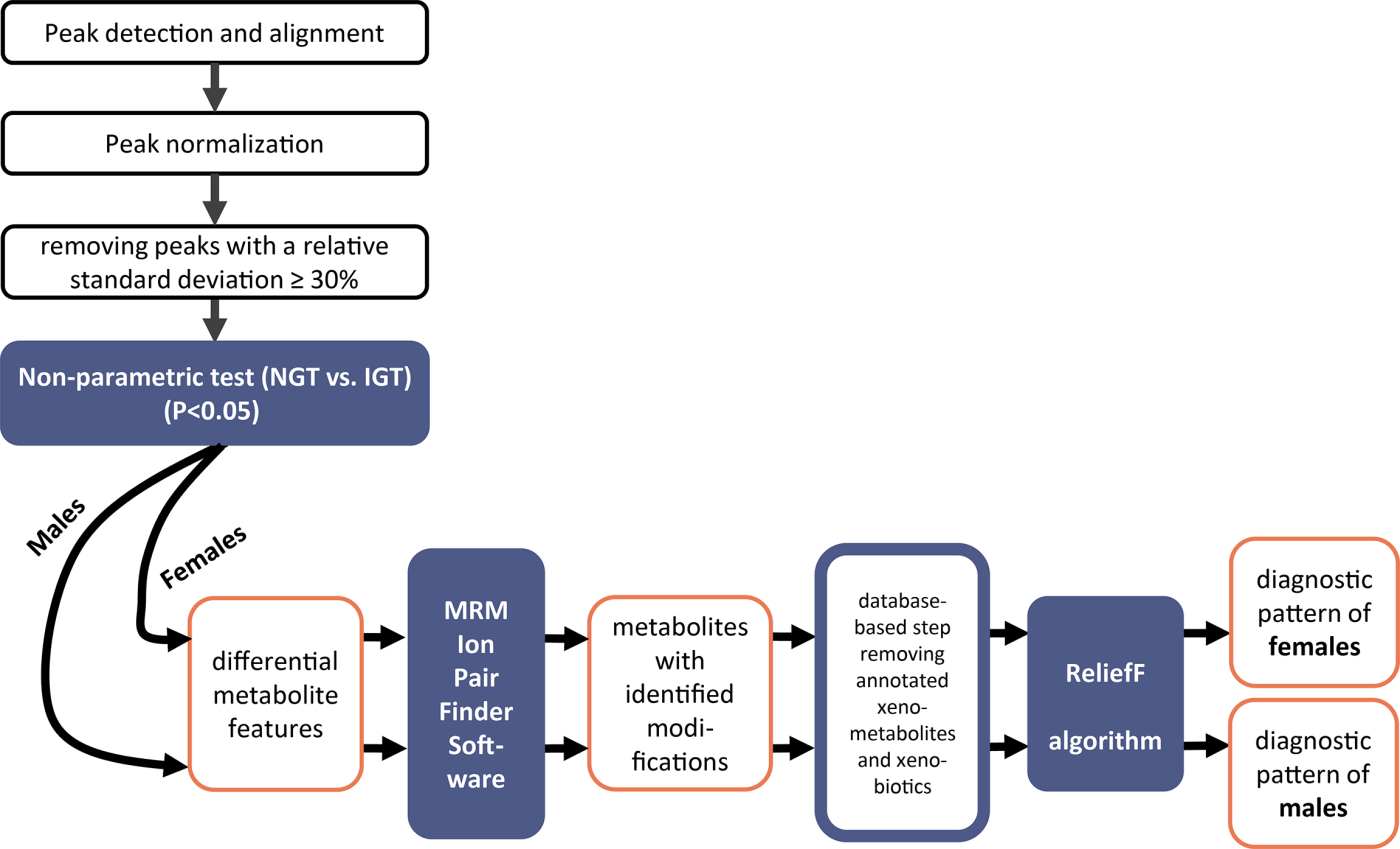
Workflow of the mass spectrometric data analysis to elucidate patterns of modified metabolites for prediabetes screening in second morning urine.

An overview of the data treatment strategy for the elucidation of a diagnostic biomarker pattern of modified metabolites is illustrated in **Figure 2**. Tolerance thresholds for mass accuracy and retention time for peak detection and alignment were related to the internal standards. Subsequent to detection and alignment of mass spectrometric data using MarkerView software (AB SCIEX, USA). The parameters for peak detection were as follows: a minimum spectral peak width of 10 ppm, a minimum RT peak width of 5 scans, and a noise threshold of 100. Retention time tolerance of 0.3 min and mass tolerance of 10 ppm were adopted for peak alignment. The features were normalized to total peak area. Only features showing relative standard deviations (RSD) <30% were kept. For the discovery of significantly different features, first non-parametric tests were applied (IBM SPSS Statistics Version 23 software). Next, MRM-Ion Pair Finder software (18) was used to exclude unmodified metabolites and extract solely metabolites with a modification in their molecular structure. Mass tolerance between precursor ion and product ion was 10 ppm. Subsequently, this biomarker list was treated further by removing annotated xenobiotics and xeno-metabolites according to HMDB database entries. Finally, diagnostic biomarker patterns in urine were elucidated by ReliefF algorithm (19). The binary logistic regression models were built by SPSS (IBM SPSS Statistics Version 23 software). SIMCA-P 11.0 software was adopted for multivariate principal component analysis (PCA) analysis using UV scaling.

## Results

### Study Design and Cohorts

**Figure 1** gives an overview of the study design, and **Table 1** shows anthropometric and clinical characteristics of NGT and IGT individuals of the discovery and the validation cohorts either as one joint cohort of female and male subjects or both sexes separately. NGT and IGT groups were matched for age and BMI. Individuals with impaired fasting glucose (IFG) only were not included, since reliable diagnosis of IFG is possible by easy, simple-to-perform analysis of FPG. Per definition, IGT subjects had higher OGTT glucose levels at 120 min, as well as fasting glucose, insulin levels, and HbA_1c_ levels compared to the joint NGT group (**Table 1**). When separated into female and male subjects, similar differences in prediabetic parameters were obtained (**Table 1**). We used the second morning urine as sample material for our metabolome screening approach, since it is less influenced by diet compared to the first morning urine (20) and sampling is not as cumbersome and error-prone as 24-h collection urine (21). Total protein content and creatinine in the second morning urine were not different between NGT and IGT (**Table 1**).

### Joint Discovery Cohort Composed of Female and Male Subjects: Detection of a Diagnostic Biomarker Pattern of Modified Metabolites for Prediabetes Screening in Second Morning Urine

The stepwise evaluation strategy of the metabolomics data applied for the joint and the sex-separated cohorts of female and male subjects to elucidate a diagnostic pattern for IGT is illustrated in a workflow scheme shown in **Figure 2**.

In the joint discovery cohort composed of female and male subjects, the non-parametric test revealed 1,296 significantly different metabolite features between NGT and IGT. Subsequently, after applying the MRM-Ion Pair Finder software (15) to extract modified metabolites from these potential biomarkers, 299 remained. Principal component analysis (PCA) revealed a clustering of IGT and NGT based on these 299 modified metabolite features (**Supplementary Figure 1A**). We proceeded by applying a feature selection algorithm [ReliefF (22)] to extract a diagnostic pattern for prediabetes screening. The ReliefF algorithm was adjusted to achieve the highest possible diagnostic sensitivity and, with respect to the great diversity of the polygenetic diabetes disease, we allowed a maximum of 20 modified metabolites in the diagnostic pattern. The ReliefF algorithm yielded a diagnostic pattern of 18 biomarkers in urine (**Supplementary Table 2**).

FPG, HbA_1c_, and fasting insulin showed diagnostic sensitivities between 35% and 67% (**Supplementary Figure 1B**; AUCs from 0.606 to 0.714). The combination of FPG and HbA_1c_ was not superior to FPG alone. We also tested glucose concentration in urine, as another possible routine diabetes measure, but not unexpectedly, all concentrations were within the reference range and no difference between NGT and IGT was observed.

In contrast to the common prediabetes screening parameters, the pattern of modified metabolites showed a better prediction for IGT (AUC 0.894, **Supplementary Figure 1B**), and the diagnostic sensitivity of the pattern was highest (85.0%, **Supplementary Figure 1B**). Aiming to improve the diagnostic performance by combining the pattern with HbA_1c_, a slight increase in diagnostic sensitivity (88.3%) was achieved, but the improvement of the AUC was small.

### Sex-Specific Discovery Cohorts Improve the Diagnostic Accuracy for Prediabetes Screening

Routine diagnostic laboratory parameters often consider differences between sexes. We hypothesized that similar differences might exist for modified metabolites. Thus, the data of the discovery cohort were re-evaluated after separation into female and male subjects. The discovery cohort is composed of 29 male subjects and 30 female subjects with NGT, and 30 male subjects and 30 female subjects with IGT, who were still well-matched for age and BMI (**Figure 1** and **Table 1**). With the workflow shown in **Figure 2**, significant differences in the levels of 760 and 707 metabolite features between NGT and IGT in female and male subjects were detected, respectively. Out of >700 biomarkers 118 and 81 modified metabolites were extracted by the MRM-Ion Pair Finder software in female and male subjects, respectively. Only 7 of these modified metabolites were identical between sexes, which corroborated the possible sex specificity of prediabetes biomarkers. Finally, after removing annotated xenobiotics and xeno-metabolites, 62 and 82 modified metabolite features remained in the male and female discovery cohorts, respectively. The ReliefF pattern recognition algorithm produced 20 and 18 modified metabolites for the differentiation of IGT and NGT in male and female subjects, respectively (**Table 2**). Only three biomarkers were identical in the patterns of male and female subjects.

**Table 2:** List of sex-specific biomarkers of modified metabolites in the diagnostic patterns for prediabetes screening in urine of male and female subjects (further analytical characteristics about each modified metabolite are given in **Supplementary Tables 3A, B**).

Male	Female
Pentosidine glucuronide^#^	Pentosidine glucuronide^#^
Glutamyl-lysine sulfate	Indoxyl sulfate
5-(3’,4’-dihydroxyphenyl)-gamma-valerolactone-3’-O-glucuronide^#^	5-(3’,4’-dihydroxyphenyl)-gamma-valerolactone-3’-O-glucuronide^#^
5-Phenylvaleric acid glucuronide	Suberic acid
3-Methoxy-4-hydroxyphenylethyleneglycol sulfate	Aspartyl-threonine glucuronide
Hippuric acid glucuronide	Glycyl-lysine
Cortisol glucuronide isomer ^a^	Malonylation (*m/z* 227.0225; *t*_R_ 5.27 min)
Tetrahydrocortisone glucuronide	Ribose conjugation (*m/z* 286.1018; *t*_R_ 3.96 min)
Cortisol glucuronide isomer ^b^	Glucuronidation (*m/z* 387.1649; *t*_R_ 10.63 min)
Phosphorylation (*m/z* 221.9842; *t*_R_ 1.06 min)	Acetylation (*m/z* 154.0611; *t*_R_ 3.97 min)
Carboxylation (*m/z* 325.0087; *t*_R_ 5.11 min)	Carboxylation (*m/z* 209.057; *t*_R_ 3.79 min)
Sulfation (*m/z* 366.0993; *t*_R_ 10.76 min)	Carboxylation (*m/z* 287.1508; *t*_R_ 11.47 min)
Hexose conjugation (*m/z* 436.1928; *t*_R_ 7.92 min)	Carboxylation (*m/z* 212.0733; *t*_R_ 3.73 min)
Glucuronidation (*m/z* 361.1014; *t*_R_ 9.83 min)	Sulfation (*m/z* 291.9590; *t*_R_ 4.27 min)
Glucuronidation (*m/z* 303.0724; *t*_R_ 4.25 min)	Sulfation (*m/z* 338.0683; *t*_R_ 9.23 min)
Glucuronidation (*m/z* 448.1612; *t*_R_ 11.66 min)^#^	Glucuronidation (*m/z* 448.1612; *t*_R_ 11.66 min)^#^
Glucuronidation (*m/z* 325.0988; *t*_R_ 11.06 min)	Glucuronidation (*m/z* 340.1136; *t*_R_ 12.58 min)
Glucuronidation (*m/z* 305.0862; *t*_R_ 4.24 min)	Glucuronidation (*m/z* 431.2461; *t*_R_ 11.77 min)
Glucuronidation (*m/z* 448.1697; *t*_R_ 9.22 min)	
Glucuronidation (*m/z* 305.1235; *t*_R_ 11.47 min)	

# same biomarkers in the patterns of male and female subjects. The glucuronidated metabolite feature of unknown annotation shows the same chromatographic retention time and mass spectrometric characteristics.

a and b represent isomers with different retention times, which cannot be differentiated by our mass spectrometric approach.

t_*R*_ = retention time under the applied chromatographic conditions on a Discovery HS F5-3 column.

**Figure 3:**
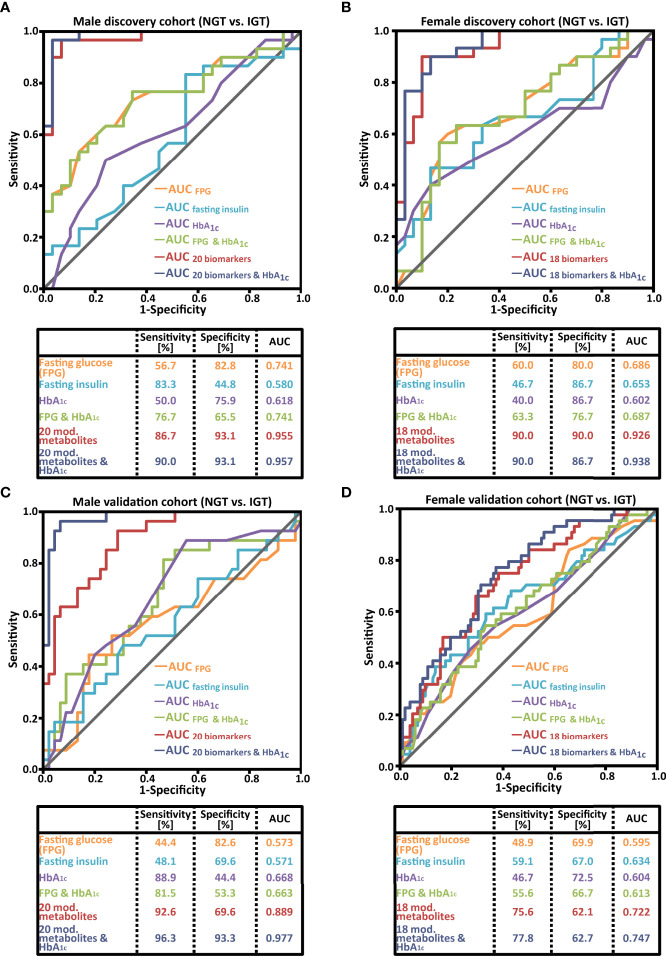
Receiver operating characteristic (ROC) curve analysis comparing the performance of a sex-specific biomarker pattern with common prediabetes laboratory parameters of **(A)** the male discovery cohort; **(B)** the female discovery cohort; **(C)** the male validation cohort; **(D)** the female validation cohort. NGT, normal glucose tolerant; IGT, impaired glucose tolerant, AUC, area under the ROC curve.

The diagnostic accuracy for prediabetes screening of the sex-specific diagnostic patterns was improved compared to the 18 biomarker patterns identified in the joint cohort of female and male subjects (**Figures 3A, B** vs. **Supplementary Figure 1B**). More importantly, the sex-specific biomarker patterns were also significantly better for IGT detection than FPG, fasting insulin, or HbA_1c_. For male subjects, the 20 combined biomarkers showed a superior AUC of 0.955 (diagnostic sensitivity of 86.7%), while the AUCs of FPG, fasting insulin, and HbA_1c_ were 0.741, 0.580, and 0.618, respectively, with diagnostic sensitivities between 50.0% and 83.3% (**Figure 3A**). In the female cohort shown in **Figure 3B**, the biomarker pattern had a discriminating ability of 0.926 (diagnostic sensitivity of 90.0%) in comparison to AUCs of FPG, fasting insulin, and HbA_1c_ of 0.686, 0.653, and 0.602, respectively (corresponding sensitivities: 60.0%, 46.7%, and 40.0%).

### Sex-Specific Validation Cohorts

As a next step, the reproducibility of the sex-specific prediabetes screening patterns was studied in second morning urine samples from the validation cohorts. For male subjects, the performance of the pattern was confirmed (**Figure 3C**; an AUC of 0.889 and a diagnostic sensitivity of 92.6%). The AUC of FPG, fasting insulin, and HbA_1c_ was 0.573, 0.571, and 0.668, respectively. The combination of the biomarker pattern and HbA_1c_ resulted in an increase of the AUC to 0.977 (**Figure 3C**), and the sensitivity and specificity reached 96.3% and 93.3%, respectively.

The pattern of female subjects, composed out of 18 biomarkers, resulted in an AUC of 0.722 (**Figure 3D**), which is better than the AUCs of FPG, fasting insulin, and HbA_1c_ (0.595, 0.634, and 0.604, respectively). The diagnostic sensitivity was 75.6%, clearly higher than the sensitivity of the three clinical parameters FPG, fasting insulin, and HbA_1c_ (49%, 59%, and 47%, respectively). For female subjects, the combination of 18 biomarkers with HbA_1c_ led only to a slight improvement in the AUC to 0.747 and sensitivity to 77.8% (**Figure 3D**).

### Annotation of Modified Metabolite Biomarkers for Prediabetes Screening

To investigate the identity of the biomarkers, MS/MS spectra and enzymatic cleavage of modifications were applied. Finally, six and nine modified metabolites in the diagnostic patterns of female and male subjects, respectively, could be named (**Table 2**). Many of these are compounds previously related to pathogenesis of diabetes or diabetic late complications, like advanced glycation end products (pentosidine-glucuronide and glutamyl-lysine-sulfate), modified metabolites from microbiota (indoxyl sulfate and dihydroxyphenyl-gamma-valerolactone-glucuronide), and hormones like glucocorticoids. Detailed chemical characteristics for all sex-specific biomarkers are provided in **Supplementary Tables 3A, B**.

## Discussion

In light of the millions of individuals with undiagnosed prediabetes worldwide, a broad screening approach is of utmost importance. However, screening for impaired glucose tolerance has not yet become feasible because reliable diagnostic tools aside from the laborious OGTT are lacking. Our biomarker discovery approach, which differed substantially from the common metabolomics strategies applied in the context of diabetes up to now, proved sex-specific patterns consisting of 18 modified metabolites in the urine of female subjects and 20 biomarkers in the urine of male subjects to be the most promising. For both sexes, these patterns proved to be significantly better in the diagnostic power for IGT screening compared to the conventional blood parameters FPG, fasting insulin, and HbA_1c_. The findings of our approach underline the potential of sex-specific prediabetes screening in urine samples.

The measurement of FPG and HbA_1c_ remains the mainstay option for state-of-the-art routine medical care screening for and diagnosis of prediabetes and diabetes, recommended by diabetes associations and public healthcare organizations worldwide (1, 23). Because FPG analysis is very simple, it is the most common approach. While being a very reliable diagnostic screening tool to detect individuals with IFG, FPG measurements miss a relevant proportion of individuals at risk to develop diabetes that have normal FPG (4–9). Data from screening studies in Europe, Asia, and America showed that approximately 30%–50% of cases were missed using FPG as the diagnostic marker (4–6). These reports are in line with the findings in our cohorts (**Figure 3**). Approaches to adjust the diagnostic threshold of HbA_1c_ (24–26) or to combine FPG and HbA_1c_ for screenings were not real breakthroughs (5). Consequently, a considerable number of individuals with prediabetes remain undiagnosed using the available and recommended diagnostic parameters for prediabetes screening.

At present, individuals at risk for diabetes can only reliably be diagnosed by measuring the concentration of blood glucose 120 min after a 75-g glucose- OGTT (4, 6). The diagnostic sensitivity and specificity of this metabolic challenge test are very high, allowing the detection of prediabetes in a reliable manner. However, this laborious, time-consuming test is unsuitable for large-scale prediabetes screening. As a consequence, for more than one decade, numerous studies aimed to discover novel biomarkers for the diagnosis of prediabetes related to the pathogenesis of T2D. Metabolomics approaches had been performed foremost in blood samples (10, 11). The biomarkers of the more than 60 reports summarized in two recent comprehensive reviews are dominated by a small number of metabolites originating from few metabolic pathways (10, 11); among these, metabolites like lysophosphatidylcholines (LPCs) and branched chain amino acids are regularly found not only in diagnostic prediabetes patterns, but also in the context of other diseases (27, 28), and therefore are not ideally suited as biomarkers for a specific disease state. To keep the diagnostic patterns for prediabetes screening simple from an analytical point of view, the majority of studies suggested a single biomarker (29, 30) or a pattern of very few metabolites (31–40). However, diabetes is a complex, polygenetic metabolic disease with a diverse pathogenesis (41). As a consequence, shifts in the circulating metabolome of subjects with prediabetes can be expected to be a combination of more general and more prediabetes subtype-specific metabolic pathway alterations. Thus, a pattern consisting of several metabolites may be more suitable for the prediabetes screening approaches than a single parameter.

Thus, we combined a metabolomics approach focused on modified metabolites with the ReliefF algorithm (42) to identify sex-specific metabolite patterns. Two patterns consisting of 18 modified metabolites for female subjects and 20 for male subjects showed the best performance. In these patterns, only three modified metabolites coincided, which underlines the sex dependency of the discovered prediabetes marker compounds. This is in line with the findings of a previous report using NMR for the analysis of urine (43). However, NMR has a much lower analytical sensitivity and, consequently, metabolite coverage than mass spectrometry. Thus, the patterns identified in this study were not superior to conventional parameters either in female or in male subjects (43). Up to now, none of the metabolomics-based studies reported reliable sex-specific biomarkers or patterns for prediabetes diagnosis (29, 31–38, 43). On the other hand, sex-specific differences, e.g., reference ranges of metabolites and other diagnostic parameters, are commonly considered in daily clinical routine (44). Thus, our results also show that there is a general need for re-thinking in the field of mass spectrometric-driven prediabetes biomarker discovery studies, considering sex-specific approaches to improve diagnostic accuracy.

Both patterns showed better diagnostic sensitivity and AUC for prediabetes screening than the routinely used parameters FPG and HbA_1c_ or a combination of both (**Figure 3**). For male subjects, the validation confirmed a very good performance of this sex-specific pattern (92.6% diagnostic sensitivity; AUC 0.889), which was even enhanced in combination with HbA_1c_ to a sensitivity of 96.3% and an AUC of 0.977. For female subjects, the diagnostic performance in the validation cohort was superior to the common blood parameters, but clearly lower than in male subjects (**Figure 3D**). One possible explanation for this lower diagnostic performance could be the higher variability in the female metabolome caused by a greater heterogeneity in the hormonal status (45–50). In addition to variations caused by the menstrual cycle, the age of the majority of female subjects intended to be screened for IGT lies between 40 and 60 years, right in the climacteric period of life with heterogenous, marked alterations of metabolic traits. We can only speculate whether taking into account these factors could improve the discovery of one or more diagnostic metabolite patterns for female subjects with prediabetes. However, recent reports from us and others clearly show that the menstrual cycle (45–47) and menopause (48–50) affect the metabolome in the blood of female subjects.

During the pathogenesis of T2D, several alterations of the metabolism and consequently the metabolome occur, some of them known and others yet unknown, building the rationale for metabolomics-based biomarker discovery studies. Among the annotated metabolites in the diagnostic biomarker patterns of male and female subjects identified in our study are compounds like advanced glycation end products (pentosidine-glucuronide and glutamyl-lysine-sulfate), modified metabolites from microbiota (indoxyl sulfate and dihydroxyphenyl-gamma-valerolactone-glucuronide), and hormones related to carbohydrate metabolism like glucocorticoids, all of which have previously been described in the pathophysiological context of diabetes (13, 51–57). Nevertheless, the majority has not been reported in any diabetes-related diagnostic biomarker pattern up to now (10, 11). Interestingly, these biomarkers originate from distinct pathogenetic reactions, which may possibly increase the diagnostic robustness of the patterns.

Pentosidine, a glucose-derived AGE, has been associated with arterial stiffness and thickness in diabetic subjects (58), and the occurrence of diabetic nephropathy (59) and retinopathy (60). ε-(γ-glutamyl)lysine, a major protein crosslink, has been described as a marker for early-stage decline in renal function (13, 61). The detection of these AGEs as biomarkers for the prediabetic state could be a first hint that there is an early decline of vascular, metabolic, and renal health in the pathogenesis of diabetes, which underlines the clinical need for improved prediabetes screening. Phenylvaleric acid, a common gut microbial metabolite of dietary flavonoids, seems to be beneficial for β-cell function and enhancement of glucose utilization in skeletal muscle (55, 62, 63). Another bacterial product of flavonoids, in this case procyanidins, found in our patterns is dihydroxyphenyl-gamma-valerolactone, which has been reported to exert protective effects against diabetes and suggested to be a marker of microbiota dysbiosis (56, 64). Another biomarker of microbiota and human metabolism among the metabolites in our patterns is indoxyl sulfate, which is described as a cardio- and nephrotoxic compound associated with the development of kidney and vascular diseases in diabetics (65, 66). 3-Methoxy-4-hydroxyphenylethyleneglycol sulfate (MHPG-S), the best marker of central norepinephrine metabolism (67), significantly correlates with blood glucose (68) and may be associated with changes or differences in body weight (69). Suberic acid is described as a ketosis marker in urine (70), which may reflect medium-chain acyl CoA-dehydrogenase (MCAD) activity (71). To the best of our knowledge, several of these metabolites have not been described in (pre)diabetes diagnostic patterns of metabolomics approaches up to now.

A possible point of criticism is the general utility of patterns consisting of a relatively large number of metabolites for diagnostic use, due to the necessity to analyze several biomarkers for a single diagnostic purpose. However, there are three important points that indicate that such an approach could be both beneficial and feasible in the future. Firstly, diagnosing complex diseases that affect various metabolic pathways in an individually different extent may only be feasible when combining various different biomarkers. This is also reflected by the low diagnostic sensitivity of single biomarkers like blood glucose and HbA_1c_ in the context of IGT detection. Secondly, combinational markers are much more robust than a single marker or a pattern of only a few biomarkers (72, 73). Thirdly, the metabolites described here can be analyzed in a single LC-MS run. Recently a fully automatic LC-MS system, suitable for 24/7 use, was introduced into routine diagnostics (74). This instrument, which is easy to operate and does not call for specific LC-MS expertise (74), not only opens up perspectives for every routine diagnostic laboratory to apply LC-MS, but also analyzes complex diagnostic patterns in a fully automatic, high-throughput manner in the future.

A potential weakness with respect to the data presented here is the relatively low number of male individuals in the validation cohort. On the other hand, the pattern performed even superior in the male validation than in the discovery cohort. Furthermore, we cannot exclude that sex hormones are among the metabolites in the biomarker pattern of female subjects for which only the modification, exact mass, and retention time (but not the identity) are known up to now. This may, in addition to the potential effects of menstrual cycle or climacteric stage, be another underlying reason for the weaker separation power of NGTs and IGTs in the female cohorts.

To conclude, a sex-specific, mass-spectrometry-driven modified metabolomics approach may greatly advance the discovery of novel biomarker patterns, at least in the context of metabolic diseases. All annotated biomarkers in the prediabetes patterns in our study showed a meaningful association to the pathogenesis of diabetes. The diagnostic performance of the sex-specific modified metabolite patterns in second morning urine to detect IGT was superior to all currently used blood parameters suitable for large-scale prediabetes screening. Together with the recent introduction of fully automated LC-MS systems, our results indicate that sex-specific metabolite patterns may greatly advance the diagnosis and, thus, treatment and counseling of subjects with prediabetes in the future.

## Data Availability Statement

The original contributions presented in the study are included in the article/**Supplementary Material**. Further inquiries can be directed to the corresponding authors.

## Ethics Statement

The studies involving human participants were reviewed and approved by ethics committee of the University of Tuebingen. The patients/participants provided their written informed consent to participate in this study.

## Author Contributions

ZL, GX, and RL designed the study. LF and AF recruited participants. LF and MH collected and prepared the samples. ZL, XYL, XZ, and PL performed the mass spectrometric metabolomics investigations. YZ, ZL, SZ, XYL, and LZ evaluated the data. AB, AF, XHL, and AP contributed to the conception and design of the study. ZL, RL, CW, XZ, XHL, and GX designed experiments, interpreted data, and wrote the manuscript. GX and RL are responsible for the integrity of the work as a whole. All listed authors contributed to drafting the manuscript or revised it critically and approved the final version.

## Funding

This study was supported by the Mobility Programme of the Sino-German Center for Research Promotion (M-0257), Chinese Academy of Sciences (CAS)-President´s International Fellowship Initiative (Grant No. 2019VBA0038), the Foundation from the Youth Innovation Promotion Association CAS (2021186), the Innovation Program (DICP ZZBS201804, DICP I201918, and DICP I202019) of Science and Research from the DICP, and the German Federal Ministry of Education and Research (BMBF) to the German Centre for Diabetes Research (Grant no. 01GI0925).

## Conflict of Interest

The authors declare that the research was conducted in the absence of any commercial or financial relationships that could be construed as a potential conflict of interest.

## Publisher’s Note

All claims expressed in this article are solely those of the authors and do not necessarily represent those of their affiliated organizations, or those of the publisher, the editors and the reviewers. Any product that may be evaluated in this article, or claim that may be made by its manufacturer, is not guaranteed or endorsed by the publisher.
