# Supplementary material for: Diagnostic Performance of Sex-Specific Modified Metabolite Patterns in Urine for Screening of Prediabetes
Source: Front Endocrinol (Lausanne). 2022 Jul 14;13:935016. doi: 10.3389/fendo.2022.935016 (PMC9333093; doi:10.3389/fendo.2022.935016)
Supplement: Supplementary file 1 [file DataSheet_1.pdf]

Supplementary Material

Supplementary Data

Annotation of modified metabolites by searching Compound Discovery 3.0 database

Compound Discovery 3.0 software (Thermo Fisher Scientific, U.S.A.) provides an integrated solution for molecule structure annotation. The MS/MS spectra acquired by Q Exactive HF full MS/data-dependent (ddMS^2) mass spectrometric analysis were used for Compound Discovery 3.0 database searching. The full MS scan parameters were as follows: mass scan range of *m/z* 50-1000, mass resolution 120000 at *m/z* 200, AGC target 3×10^6, maximum IT 100 ms, capillary temperature 320 °C, aux gas heater temperature 350 °C, sheath gas 45 (arbitrary units), aux gas 10 (arbitrary units), and S-lens RF level 50. The spray voltage was 3.5 kV in positive ion mode and 3.0 kV in negative ion mode. The MS/MS scan parameters were as follows: AGC target 1×10^5, maximum IT 50 ms, isolation windows of 1.0 *m/z*. Fragmentation was performed at mixed normalized collision energies of 15%, 30% and 45%. MS/MS acquisition was triggered by the top 10 most abundant ions based on the inclusion list. To collect MS/MS for as many precursor ions as possible, the mass range was divided into four segments according to the number of ions in the full scan. The total mass range was *m/z* 50-1000. A loop count of 2 was used for each segment. Once the MS/MS of an ion was obtained, it was excluded from the second loop.

Supplementary Figures and Tables

Supplementary Figures

A

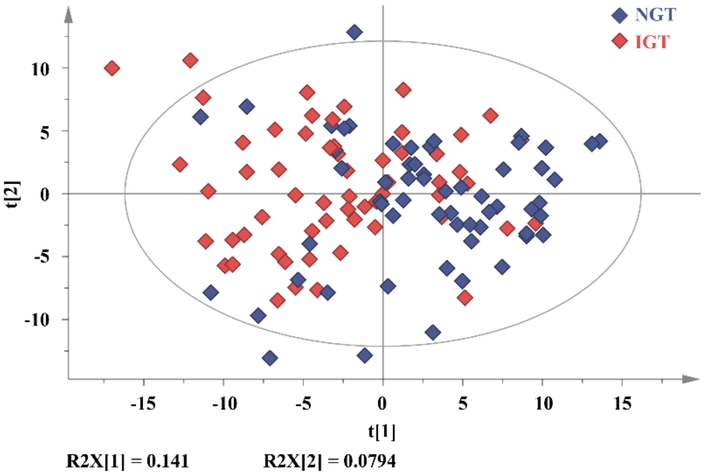

B

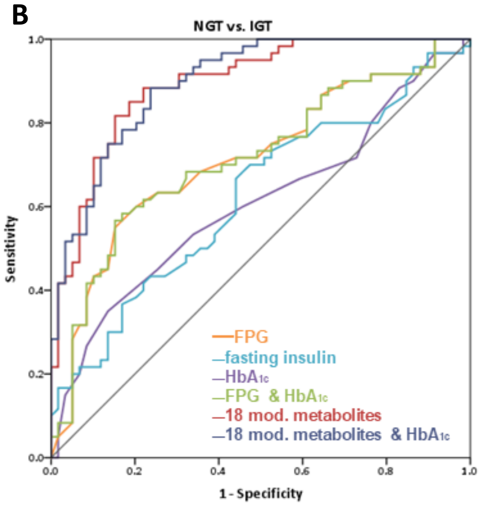

	Sensitivity [%]	Specificity [%]	AUC
Fasting glucose (FPG)	56.7	83.1	0.714
Fasting insulin	66.7	55.9	0.623
HbA_{1c}	35.0	86.4	0.606
FPG & HbA_{1c}	58.3	83.1	0.715
18 mod. metabolites	85.0	81.4	0.894
18 mod. metabolites & HbA_{1c}	88.3	76.3	0.897

Supplementary Figure 1. (A) Scores plot of a principal component analysis (PCA) of 299 modified metabolites in second morning urines of normal glucose tolerant (NGT; blue colour) and impaired glucose tolerant (IGT; red colour) individuals of the joint discovery cohort of females and males; (B) Diagnostic performance of a biomarker pattern of 18 modified metabolites (see ESM Table 2 for details about the metabolites) for the detection of individuals having impaired glucose tolerance in comparison to common routine parameters in the joint discovery cohort of females and males.

Supplementary Tables

Supplementary Table 1. List of profiled fragment ions to detect modifying structures.

Modification type	Molecular formula	Exact mass	Ref.
Methylation	CH_2	14.0157	(1)
Ammonia	NH_3	17.0260	(2)
Methyl ammonia	CH_3NH_2	31.0417	(3)
Acetylation	CH_2CO	42.0106	(4)
Carboxylation	COO/HCOOH	43.9893 / 46.0049	(2, 3)
Glycine conjugation	$C_2H_3O_2NH_2$	75.0320	(5)
Sulfation	SO_3	79.9568	(6)
Malonylation	$C_3H_2O_3$	85.9999	(7)
Phosphorylation	H_3PO_4	97.9769	(8)
Ribose conjugation	$C_5H_8O_4$	132.0423	(2)
Anhydrodeoxyhexose conjugation	$C_6H_{10}O_4$	146.0574	(3)
Hexose conjugation	$C_6H_{10}O_5$	162.0528	(9)
Glucuronidation	$C_6H_8O_6$	176.0321	(10)
Glucuronic acid conjugation	$C_6H_{10}O_7$	194.0421	(3)
Palmitic acid conjugation	$C_{16}H_{30}O$	238.2297	(2)

Supplementary Table 2. Detailed analytical information about the 18 biomarkers in the pattern of the joint discovery cohort of females and males for pre-diabetes screening in second morning urine.

Ion mode	m/z	Ret. Time (min)	Compound	Neutral loss	Adduct	Database
neg	269.1528	9.43	carboxylation[b]	COO		
pos	300.1543	6.26	ribose conjugation[b]	$C_5H_8O_4$		
neg	391.1881	11.80	3-Hydroxydodecanoic acid glucuronide[a,b]	$C_6H_8O_6$	M-H	MyCompoundID
pos	361.2225	13.01	glucuronidation[b]	$C_6H_8O_6$		
pos	291.1199	12.49	carboxylation[b]	COO		
pos	375.0535	1.22	glucuronidation[b]	$C_6H_8O_6$		
pos	181.0857	9.94	carboxylation[b]	COOH		
pos	271.1649	9.32	anhydrodeoxyhexose conjugation[b]	$C_6H_{10}O_4$		
pos	271.1635	10.24	ammonia[b]	NH_3		
neg	436.2254	10.99	glucuronidation[b]	$C_6H_8O_6$		
neg	225.0635	2.58	5-acetamido-6-formamido-3-methyluracil[a,b,c]	CH_2CO	M-H	Compound Discovery
neg	405.0484	5.55	glucuronidation[b]	$C_6H_8O_6$		
neg	439.0634	10.13	glucuronidation[b]	$C_6H_8O_6$		
neg	283.0280	8.25	3-Methoxy-4-hydroxyphenylethyleneglycol sulfate[a,b]	SO_3	M+F	HMDB
neg	379.1017	9.41	5-(3',4'-dihydroxyphenyl)-gamma-valerolactone-3'-O-glucuronide[a,b]	$C_6H_8O_6$	M-H_2O-H	HMDB
neg	511.2551	11.70	3-alpha-hydroxy-5-alpha-androstane-17-one 3-D-glucuronide[a,b]	$C_6H_8O_6$	M+FA-H	HMDB
neg	539.2469	11.35	Tetrahydroaldosterone-3-glucuronide[a,b]	$C_6H_8O_6$	M-H	HMDB
neg	287.1508	11.47	carboxylation[b]	COO		

a) based on exact mass;b) based on the modification group found by MRM-Ion Pair Finder software; c) based on the fragmentation pattern

Supplementary Table 3A. Detailed analytical information about sex-specific biomarkers in the pattern to perform pre-diabetes screening in urine of males.

Ion mode	m/z	Ret. Time (min)	Compound	Neutral loss	Adduct	Database
neg	478.1697	9.22	glucuronidation[b,d]	$C_6H_8O_6$		
neg	353.1234	13.67	5-Phenylvaleric acid glucuronide[a,b,d]	$C_6H_8O_6$	M-H	MyCompoundID
neg	354.0827	5.9	Hippuric acid glucuronide[a,b,c,d]	$C_6H_8O_6$	M-H	Compound Discovery
neg	553.2251	14.67	Pentosidine glucuronide[a,b,d]	$C_6H_8O_6$	M-H	MyCompoundID
neg	303.0724	4.25	glucuronidation[b,d]	$C_6H_8O_6$		
neg	325.0988	11.06	glucuronidation[b]	$C_6H_8O_6$		
neg	448.1612	11.66	glucuronidation[b,d]	$C_6H_8O_6$		
pos	539.2454	11.5	Cortisol glucuronide[a,b,c,d,*]	$C_6H_8O_6$	M+H	Compound Discovery
pos	541.2627	11.88	Tetrahydrocortisone glucuronide[a,b,c,d]	$C_6H_8O_6$	M+H	Compound Discovery
pos	305.0862	4.24	glucuronidation[b,d]	$C_6H_8O_6$		
neg	305.1235	11.47	glucuronidation[b,d]	$C_6H_8O_6$		
pos	539.2440	11.01	Cortisol glucuronide [a,b,c,d,*]	$C_6H_8O_6$	M+H	Compound Discovery
pos	361.1014	9.83	glucuronidation[b]	$C_6H_8O_6$		
neg	379.1017	9.41	5-(3',4'-dihydroxyphenyl)-gamma-valerolactone-3'-O-glucuronide[a,b]	$C_6H_8O_6$	M-H_2O-H	HMDB
pos	436.1928	7.92	hexose conjugation[b]	$C_6H_{10}O_5$		
neg	221.9842	1.06	phosphorylation[b]	H_3PO_4		
neg	325.0087	5.11	carboxylation[b]	HCOOH		
neg	366.0993	10.75	sulfation[b,d]	SO_3		
pos	356.1159	9.27	Glutamyl-Lysine sulfate[a,b,d]	SO_3	M+H	MyCompoundID
neg	283.0280	8.25	3-Methoxy-4-hydroxyphenylethyleneglycol sulfate[a,b]	SO_3	M+F	HMDB

a) based on exact mass; b) based on the modification group found by MRM-Ion Pair Finder software; c) based on the fragmentation pattern; d) modification identity confirmed by enzymatic cleavage; * isomers

Supplementary Table 3B. Detailed analytical information about sex-specific biomarkers in the pattern to perform pre-diabetes screening in urine of females.

Ion mode	*m/z*	Ret. Time (min)	Compound	Neutral loss	Adduct	Database
pos	227.0225	5.27	malonylation[b]	$C_3H_2O_3$		
pos	286.1018	3.96	ribose conjugation[b]	$C_5H_8O_4$		
neg	387.1649	10.63	glucuronidation[b,d]	$C_6H_8O_6$		
neg	379.1017	9.41	5-(3',4'-dihydroxyphenyl)-gamma-valerolactone-3'-O-glucuronide[a,b]	$C_6H_8O_6$	M-H_2O-H	HMDB
neg	553.2251	14.67	Pentosidine glucuronide[a,b,d]	$C_6H_8O_6$	M-H	MyCompoundID
neg	448.1612	11.66	glucuronidation[b,d]	$C_6H_8O_6$		
neg	340.1136	12.58	glucuronidation[b,d]	$C_6H_8O_6$		
pos	431.2461	11.77	glucuronidation[b,d]	$C_6H_8O_6$		
neg	409.1131	11.14	Aspartyl-threonine glucuronide[a,b,d]	$C_6H_8O_6$	M-H	MyCompoundID
pos	154.0611	3.97	Acetylation[b]	CH_2CO		
neg	209.0572	3.79	carboxylation[b]	COO		
neg	287.1508	11.47	carboxylation[b]	COO		
pos	212.0733	3.73	carboxylation[b]	HCOOH		
pos	204.1324	5.47	Glycyl-Lysine[a,b]	HCOOH	M+H	Metlin
pos	157.0844	8.93	Suberic acid[a,b,c]	HCOOH	M+H-H_2O	Compound Discovery
neg	291.9590	4.27	sulfation[b]	SO_3		
neg	212.0030	4.29	Indoxyl sulfate[a,b,d]	SO_3	M-H	HMDB
neg	338.0683	9.23	sulfation[b,d]	SO_3		

a) based on exact mass; b) based on the modification group found by MRM-Ion Pair Finder software; c) based on the fragmentation pattern; d) modification identity confirmed by enzymatic cleavage

References

1. Lee R, West D, Phillips SM, Britz-McKibbin P. Differential Metabolomics for Quantitative Assessment of Oxidative Stress with Strenuous Exercise and Nutritional Intervention: Thiol-Specific Regulation of Cellular Metabolism with N-Acetyl-L-Cysteine Pretreatment. *Analytical Chemistry* (2010) 82(7):2959-68. doi: 10.1021/ac9029746.

2. Nicholson JK, Holmes E, Kinross J, Burcelin R, Gibson G, Jia W, et al. Host-Gut Microbiota Metabolic Interactions. *Science* (2012) 336(6086):1262. doi: 10.1126/science.1223813.

3. Liu L, Wang M, Yang X, Bi M, Na L, Niu Y, et al. Fasting Serum Lipid and Dehydroepiandrosterone Sulfate as Important Metabolites for Detecting Isolated Postchallenge Diabetes: Serum Metabolomics Via Ultra-High-Performance Lc-Ms. *Clin Chem* (2013) 59(9):1338-48. doi: 10.1373/clinchem.2012.200527.

4. Gouveia SC, Castilho PC. Characterization of Phenolic Compounds in Helichrysum Melaleucum by High-Performance Liquid Chromatography with on-Line Ultraviolet and Mass Spectrometry Detection. *Rapid Commun Mass Spectrom* (2010) 24(13):1851-68. doi: 10.1002/rcm.4585.

5. Stensballe A, Jensen ON, Olsen JV, Haselmann KF, Zubarev RA. Electron Capture Dissociation of Singly and Multiply Phosphorylated Peptides. *Rapid Communications in Mass Spectrometry* (2000) 14(19):1793-800. doi: 10.1002/1097-0231(20001015)14:19<1793::AID-RCM95>3.0.CO;2-Q.

6. Glauser G, Boccard J, Rudaz S, Wolfender JL. Mass Spectrometry-Based Metabolomics Oriented by Correlation Analysis for Wound-Induced Molecule Discovery: Identification of a Novel Jasmonate Glucoside. *Phytochem Anal* (2010) 21(1):95-101. doi: 10.1002/pca.1155.

7. Wen H, Yang HJ, An YJ, Kim JM, Lee DH, Jin X, et al. Enhanced Phase Ii Detoxification Contributes to Beneficial Effects of Dietary Restriction as Revealed by Multi-Platform Metabolomics Studies. *Molecular & cellular proteomics : MCP* (2013) 12(3):575-86. doi: 10.1074/mcp.M112.021352.

8. Fischer CR, Wilmes P, Bowen BP, Northen TR, Banfield JF. Deuterium-Exchange Metabolomics Identifies N-Methyl Lyso Phosphatidylethanolamines as Abundant Lipids in Acidophilic Mixed Microbial Communities. *Metabolomics* (2012) 8(4):566-78. doi: 10.1007/s11306-011-0344-x.

9. Li L, Li R, Zhou J, Zuniga A, Stanislaus AE, Wu Y, et al. Mycompoundid: Using an Evidence-Based Metabolome Library for Metabolite Identification. *Anal Chem* (2013) 85(6):3401-8. doi: 10.1021/ac400099b.

10. Levsen K, Schiebel HM, Terlouw JK, Jobst KJ, Elend M, Preiss A, et al. Even-Electron Ions: A Systematic Study of the Neutral Species Lost in the Dissociation of Quasi-Molecular Ions. *J Mass Spectrom* (2007) 42(8):1024-44. doi: 10.1002/jms.1234.
